# Acute contact appendicitis due to a migrated pericardial drain: a case report

**DOI:** 10.4076/1757-1626-2-6250

**Published:** 2009-07-30

**Authors:** Daniel Paramythiotis, Theodossis S Papavramidis, Vassilis N Papadopoulos, Antonis Michalopoulos, Olia Vasilaki, Nick Harlaftis

**Affiliations:** 1 1st Propedeutic Surgical Clinic, A.H.E.P.A. University Hospital, Aristotle’s University of ThessalonikiThessalonikiGreece; 2 Department of Microbiology, A.H.E.P.A. University Hospital of Thessaloniki, Aristotle’s University of ThessalonikiThessalonikiGreece

## Abstract

**Introduction:**

The literature is replete with articles of foreign-body appendicitis and periappendicitis, but to our knowledge there are only two reports of extraintestinal foreign bodies causing contact appendicitis.

**Case presentation:**

A 47-year old woman presented to the emergency department with a 24-hour history of right iliac fossa pain, nausea and vomiting, high fever and palpable right iliac fossa mass. The patient had an anamnestic of systemic lupus erythematosus, that caused acute pericarditis with effusion, that was treated with pericardiotomy and a pericardial drain. The laboratory tests showed leukocytosis The plain abdomen film showed no radiologic signs corresponding to acute abdomen, while the computed tomography revealed a radio-opaque formation in the right iliac fossa, corresponding to the palpable mass. Exploratory laparotomy revealed a pericardial drain. The microbiologic analysis of the abscess revealed *Salmonella*. The postoperative course of the patient was uneventful.

**Conclusion:**

Acute appendicitis due to a foreign body, without an anamnestic of either surgery or injury may cause a severe diagnostic dilemma. The computed tomography images may lead to logic riddles that have to be solved by an explorative laparotomy. Foreign bodies rarely cause acute abdomen, nevertheless the probability has to be considered when an interventional technique has been applied even if the location of the intervention is far from the abdominal cavity.

## Introduction

The literature is replete with articles of foreign-body appendicitis and periappendicitis, including three comprehensive review articles on this topic [1-3]. These articles have reported many types of ingested foreign bodies within the appendix. Klinger et al reported that ingested foreign bodies account for the 0.0005% of the aetiology of acute appendicitis [1]. Hard pointed objects such as toothpicks, pins, needles, nails, and screws are prone to causing perforation, but other foreign bodies —some seemingly innocuous— such as thread, chewing gum, and hair have been reported as the likely causes of appendicitis resulting from obstruction, as have lodged bullets, ingested lead shot, and parasitic worms topic [1-3].

To our knowledge there are only two reports of extraintestinal foreign bodies causing appendicitis [4,5]. The present case is extremely interesting because the appendicitis is caused by an extraintestinal foreign body which originated from the thoracic cavity. Furthermore, this is the only case in the litterature that a pericardial drain causes acute abdomen.

## Case presentation

A 47-year old Greek woman presented to the emergencies with a 24 hour history of right iliac fossa pain, nausea and vomiting, high fever and palpable right iliac fossa mass. The patient had an anamnestic of systemic lupus erythematosus, that caused three years ago acute pericarditis with effusion, which was treated with pericardiotomy and placement of a pericardial drain.

**Figure 1.**
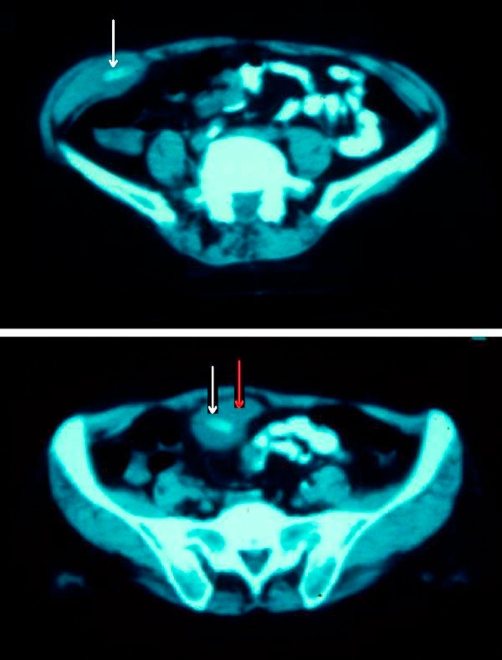
CT showing the localisation of the drain that migrated in the right iliac fossa. White arrow indicates the drain, while red arro indicates the abscess.

Laboratory tests showed leukocytosis (15.100 WBC/*μ*L with 86% polymorphs). The plain abdomen film showed no radiologic signs corresponding to acute abdomen, while the CT revealed a radio-opaque formation in the right iliac fossa, corresponding to the palpable mass (Figure 1).

Exploratory laparotomy revealed a foreign body (8 cm long) corresponding to the pericardial drain. The drain had migrated to the right iliac fossa causing the formation of a periappendiceal abscess. The microbiologic analysis of the abscess revealed Salmonella. The postoperative course of the patient was uneventful.

## Discussion

Pericardial disorders occurring in connective tissue diseases are not uncommon and may present as acute or chronic pericarditis with or without an effusion. In many instances, a diagnosis of pericardial involvement is not found until autopsy [6]. Furthermore, pericarditis is the most common cardiac abnormality in systemic lupus erythematosus (SLE) patients [7]. Our patient presented, three years before the acute appendicitis, pericarditis due to SLE.

The drainage of pericardial effusion is an established practice concomitant to surgical pericardiotomy and pericardiectomy [8]. Our patient had a pericardial drain connecting the pericardial cavity to the abdominal cavity in order to evacuate the pericardial effusion.

Salmonella was isolated from the periappendiceal abscess. The fact that this intestinal pathogen was involved in the acute abdomen formation has to let us consider the probability of its being the primary pathology. We have at least to aknowledge the fact that it is possible that the Salmonella infection led to intestinal perforation and that the drain was an incidental finding.

## Conclusion

Acute appendicitis due to a foreign body, without an anamnestic of either surgery or injury may cause a severe diagnostic dilemma. The CT images may lead to logic riddles that have to be solved by an explorative laparotomy. Foreign bodies rarely cause acute abdomen, nevertheless the probability has to be considered when an interventional technique has been applied even if the location of the intervention is far from the abdominal cavity.

## Abbreviations

CT: computed tomography
SLE: systemic lupus erythematosus
WBC: white blood cells
